# AI based rehabilitation: the way forward in addressing unmet needs in musculoskeletal disease

**DOI:** 10.3389/fpubh.2026.1773733

**Published:** 2026-02-25

**Authors:** João Paulo Branco, Duarte Tude Graça, Eduardo Costa, Catarina Aguiar Branco, Francisco Sampaio, João Barroso, João Páscoa Pinheiro, Pedro Cantista, Renato Nunes, Jorge Lains

**Affiliations:** 1 Physical and Rehabilitation Medicine Department, ULS Coimbra, Coimbra, Portugal; 2 Faculty of Medicine, University of Coimbra, Coimbra, Portugal; 3 Portuguese Society of Physical and Rehabilitation Medicine (SPMFR), Lisbon, Portugal; 4 Faculdade de Medicina, Universidade de Lisboa, Lisbon, Portugal; 5 Centre for Public Administration and Public Policies, Institute of Social and Political Sciences, University of Lisbon, Lisbon, Portugal; 6 Centre for Management Studies (CEGIST), Instituto Superior Técnico, Universidade de Lisboa, Lisbon, Portugal; 7 Physical and Rehabilitation Medicine Department, Entre o Douro e Vouga Local Health Unit, Santa Maria da Feira, Portugal; 8 Faculty of Dental Medicine, University of Porto, Porto, Portugal; 9 Physical and Rehabilitation Medicine College, Portuguese Medical Association, Lisbon, Portugal; 10 Professional Practice Committee, UEMS PRM Section and Board, Brussels, Belgium; 11 European Society of Physical and Rehabilitation Medicine (ESPRM), Zagreb, Croatia; 12 Physical and Rehabilitation Medicine Department, ULS Santa Maria, Lisbon, Portugal; 13 Faculty of Medicine, Physical and Rehabilitation Medicine University Clinic, University of Lisbon, Lisbon, Portugal; 14 Physical and Rehabilitation Medicine Department, ULS São João, Porto, Portugal; 15 Faculty of Medicine, University of Porto, Porto, Portugal; 16 Physical and Rehabilitation Medicine Department, ULS Santo António, Porto, Portugal; 17 School of Medicine and Biomedical Sciences, University of Porto, Porto, Portugal; 18 Physical and Rehabilitation Medicine Department, Hospital da Prelada, Porto, Portugal; 19 Stroke Rehabilitation Unit, Hospital da Prelada, Porto, Portugal; 20 Department of Neuromusculoskeletal Surgery and Rehabilitation, ULS Coimbra, Coimbra, Portugal; 21 Centro de Medicina de Reabilitação da Região Centro Rovisco Pais, ULS Coimbra, Tocha, Portugal; 22 European Academy of Rehabilitation Medicine (EARM), Brussels, Belgium

**Keywords:** AI-based rehabilitation, artificial intelligence, health system efficiency, musculoskeletal disorders, rehabilitation access

## Abstract

Musculoskeletal (MSK) conditions are the leading cause of disability worldwide and, in European Union countries, account for up to 17% of years lived with disability and around 2% of gross domestic product (GDP) in direct and indirect costs. Despite universal health coverage and a doubling of public rehabilitation prescriptions in the past decade, unmet rehabilitation needs persist in Portugal, alongside growing regional disparities, long waiting times, and a heavy reliance on private services for physical rehabilitation. These factors undermine both clinical and economic outcomes. International and national evidence indicates that rehabilitation delivered through AI-enabled programmes is feasible and potentially effective, can be deployed at scale, and may reduce barriers related to geography, scheduling, and limited rehabilitation facilities. Such solutions may help improve continuity of care, shorten waiting times, and address unmet needs, but large-scale adoption requires robust frameworks for clinical evaluation and validation, patient selection, professional training, and outcome monitoring, often within hybrid models of care. By explicitly addressing potential benefits, risks, limitations, and clinical criteria, the rehabilitation community can facilitate responsible and ethical integration of AI-supported and digital models into rehabilitation practice and research, while managing the organisational and cultural changes needed to incorporate these models as complementary interventions within health systems. Drawing on WHO and OECD recommendations and on recent Portuguese implementation experience, this perspective examines how AI-driven rehabilitation may support more equitable, timely, and efficient responses to MSK rehabilitation needs, particularly for physician-prescribed care delivered under medical supervision in the home setting.

## Introduction

The World Health Organization (WHO) defines rehabilitation as “a set of interventions designed to optimize functioning and reduce disability in individuals with health conditions in interaction with their environment” (1). This person-centred approach aims to enable individuals to remain independent and participate in meaningful roles despite health conditions (1). Globally, an estimated 2.4 billion people live with conditions that would benefit from rehabilitation, a need that has increased by 63% since 1990 and is now a core component of universal health coverage (2).

Although more than half of the population lacks adequate access to rehabilitation services, the Portuguese context is heterogeneous. Despite a structured public and private network, limitations persist, including unequal distribution of services across regions, significant waiting lists, human resource constraints, and the incipient adoption of telerehabilitation solutions (3). As the leading contributor to disability worldwide, 1.71 billion people live with Musculoskeletal (MSK) conditions, which account for 17% of all years lived with disability (YLDs) and approximately two-thirds of adults in need of rehabilitation (4).

Beyond clinical burden, MSK imposes substantial economic costs. Across OECD countries, MSK conditions account for 17.4% of total health expenditure, the highest proportion of any disease category (5). In the European Union, MSK disorders cost approximately 2% of GDP driven by healthcare expenditures, work absences, and productivity losses and are a leading cause of absenteeism, presenteeism, and productivity loss in high-income countries, accounting for up to 60% of work-related absences and over 130 million lost workdays annually across the OECD (6, 7).

In 2005, the WHO approved resolution WHA58.23 (“Disability, including prevention, management and rehabilitation”), formally recognising rehabilitation as part of the obligations of Member States (8). The subsequent WHA76.6 resolution “Strengthening rehabilitation in health systems,” stressed that rehabilitation is fundamental to human rights, human capital, and socio-economic development, while noting that more than 50% of people in many countries do not receive needed services (3, 9). In parallel, the European Digital Decade and the WHO Rehabilitation 2030 vision and UEMS PRM Section and Board Position Statement (10) have positioned artificial intelligence (AI) and digitally enabled care as key pathways to more equitable, scalable, and financially sustainable rehabilitation (9) while highlighting potential benefits, risks and ethical principles (10).

Ethical Principles in AI rehabilitation should follow the WHO guidance on ethics and governance of AI for health and the European Commission’s Ethics Guidelines for Trustworthy AI (11). Clinicians, AI developers and healthcare administrators should be involved throughout implementation to ensure that AI’s benefits, opportunities, and developments are achieved.

Building on this, what remains unclear is not *whether* rehabilitation access must improve, but *how,* given that many countries are unequipped. By 2030, one in six people globally will be aged 60 or older (12). By 2050, that figure will double. At the same time, MSK is projected to affect 2.16 billion people by 2035 (13). This perspective article examines AI-enabled rehabilitation as a potential pathway to address access barriers. It reviews clinical evidence, implementation experience and a Portuguese pilot, while exploring the potential in expanding access, improving efficiency and supporting value-based care.

## The rehabilitation access gap

The WHO identifies multiple access barriers to rehabilitation services, such as limited prioritization and funding; shortage of services outside urban centers; long waiting times; high out-of-pocket costs; insufficient trained professionals; inadequate resources including technology, fragmented research and data infrastructure and ineffective referral pathways (14, 15). Portugal, a European high-income country with universal health coverage, does not experience all these barriers to the same extent. The Portuguese National Health Service (NHS), established in 1979, provides universal coverage. Most patients ultimately access Physical and Rehabilitation Medicine (PRM) and other rehabilitation services without direct costs, either in public hospitals or in private clinics under contractual arrangements. However, non-financial barriers such as geographical imbalances, organizational fragmentation and waiting times still contribute to unequal access to rehabilitation (16).

Between 2011 and 2014, during the national financial crisis, the Ministry of Health established the Technical Group for Hospital Reform (GTRH). Its final report, “Citizens at the Centre of the System, Professionals at the Centre of Change,” proposed reorganising the national hospital network and promoting better articulation with private, contracted, and social sectors. In line with these recommendations, the Central Administration of the Health System (ACSS), in January 2017, published the National Network of Hospital Specialty and Referral for Physical Medicine and Rehabilitation (RNEHRMFR), designed to integrate public-sector rehabilitation into a functional network and to establish links with other providers, with the aim of ensuring equitable access to high-quality care regardless of geographic location or socioeconomic status. However, the National Health Regulation Authority (ERS) has documented marked geographic disparities, with 28.2% of Portuguese municipalities lacking any SNS-contracted PRM providers, especially in low density regions, underscoring the value of developing complementary models of care that can strengthen equity and responsiveness (17).

## High and rising costs of publicly funded rehabilitation

In addition to access gaps, the Portuguese NHS faces rising rehabilitation costs. In Portugal, a significant share of costs related to PRM is directed at private and social sector clinics, through conventions and agreements. Access to these services typically begins with an NHS physician referral to PRM, after which a PRM specialist prescribes a rehabilitation program, assesses function and frames motivation, expectations, and rehabilitation goals. Although provision of care is ensured by private providers, financing is ensured by the NHS. In fact, PRM is the fourth largest source of conventioned healthcare expenditure in the Portuguese NHS, after laboratory analyses, imaging and haemodialysis (18).

**Figure 1:**
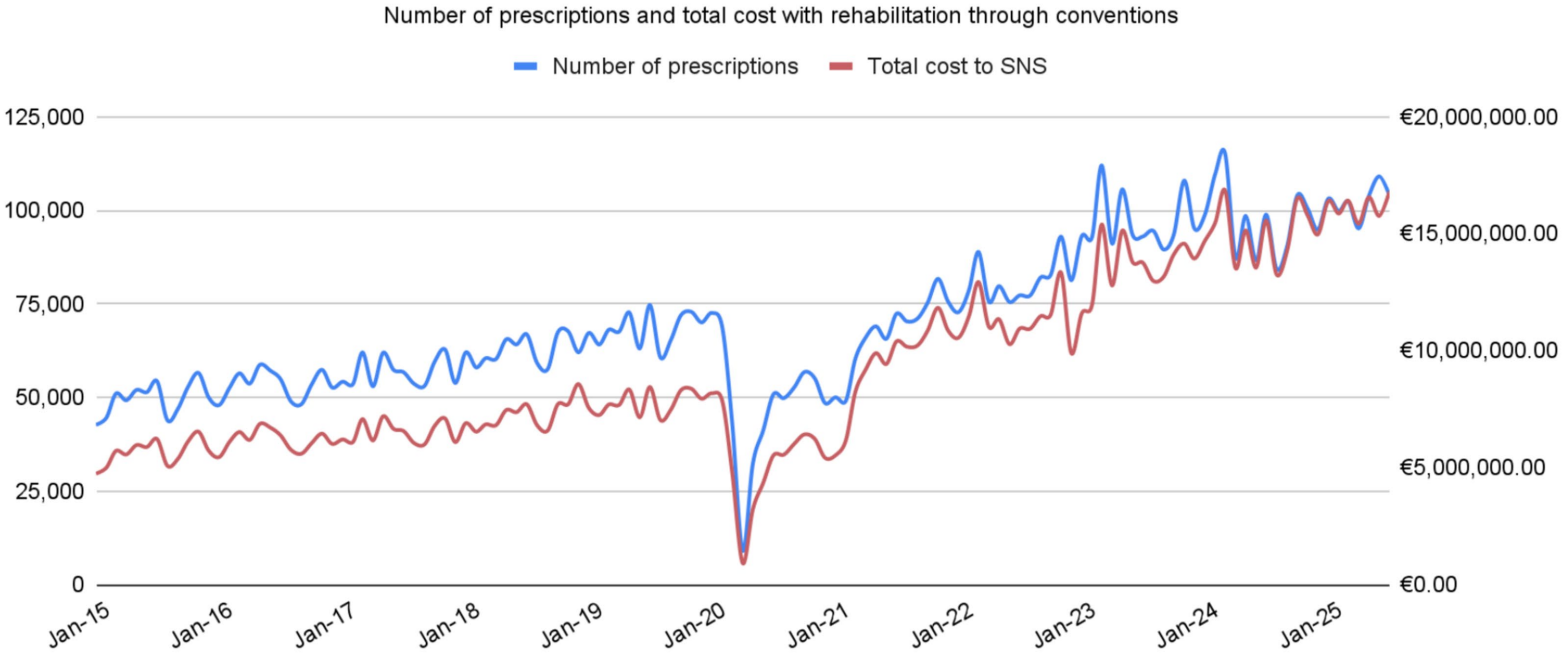
Trends in monthly rehabilitation prescriptions and total expenditure in the Portuguese NHS from 2015 to 2025. Source: Serviço Nacional de Saúde (19).

Over the past decade, there was a clear and consistent trajectory of increased utilization of these services, except during the COVID pandemic. The average number of monthly prescriptions doubled from approximately 50,000 per month in 2015 to roughly 100,000 in the first 7 months of 2025, and associated costs have risen even more sharply (see Figure 1). Expenditure in the first 7 months of 2025 (around €113 M) has already surpassed, and almost doubled, the total annual cost recorded in 2015 (€67 M), while total spending in 2024 was close to €180 M (19).

Out of the total cost of rehabilitation, a significant share is related to MSK conditions and their treatment (20). Evidence from a Portuguese primary care setting indicates that approximately 70% of PRM referrals are associated with MSK conditions, the majority of rehabilitation activity in that context (18, 20). National reports on contracted PRM describe case-mix profiles consistent with a predominance of MSK-related problems, although expenditure is not systematically stratified by diagnosis. In the absence of more granular national data on costs by diagnostic group, this article assumes a conservative scenario range in which MSK conditions are assumed to represent between 50 and 70% of total physical rehabilitation expenditure. This range should be understood as an approximation used to frame exploratory economic scenarios, and its limitations, namely, including the fact that it is partly extrapolated from referral volumes rather than direct cost data, are acknowledged.

Considering the assumption above and using the 2024 yearly cost as a baseline (€180 M), we estimate that roughly €90–126 M of contracted rehabilitation expenditure could be attributable to MSK conditions. The growth in prescriptions generates alarming second-order effects that go beyond the increasing cost burden. As demand grows, providers under the convention agreements may be unable to scale capacity proportionally, which in turn may lead to higher lead times and waiting lists, negatively affecting treatment outcomes. These pressures call for further development of complementary in-person and telerehabilitation approaches, as well as adapting clinical, organizational and financial models in both public and private units so that access, continuity and efficiency improve.

## The Portuguese reality

A report from the Executive Board of the National Health Service (DE-SNS) positions digital transformation as a key priority for the country’s healthcare system, highlighting key developments (21). Within this agenda, AI-based telerehabilitation is introduced as one of the hallmark initiatives, with pilot projects suggesting the potential gains in access, efficiency and patient experience (21).

A growing body of evidence supports AI-enabled rehabilitation solutions as feasible, safe, and effective alternatives to in-person care, including data generated in Portugal (22). In Portugal, the results from the implementation of an AI-based rehabilitation solution in a university-affiliated hospital and two large primary care clusters in the metropolitan Porto region have been of particular interest. This pilot involved a leading hospital (*Centro Hospitalar Universitário São João*) and regional networks of publicly funded primary healthcare centres serving the populations of regions from Oporto city. Launched in December 2023, the pilot included post-operative patients, specifically individuals recovering from rotator cuff repair, hip or knee arthroplasty, and minimally invasive ultrasound-guided shoulder procedures, as well as chronic MSK patients. All patients underwent clinical assessment, treatment planning, and ongoing supervision by PRM specialists, with many following hybrid pathways combining digital and in-person rehabilitation.

The platform combined AI technologies with clinical expertise to prevent and treat pain, while reducing barriers related to access and costs. The patient pathway included initial contact and assessment, medical device delivery within 48 h, telerehabilitation delivery, and progress reports sent to prescribing PRM specialists every 2–4 weeks. Additionally, the digital solution provided structured exercise guidance with real-time feedback on exercise performance. Patients had 24-h PRM access and periodic re-evaluation every 4–8 weeks depending on condition, with final assessment and discharge when protocol completion and functional targets were achieved. Treatment protocols were: rotator cuff repair (16–20 weeks); hip and knee arthroplasty (8 weeks); adhesive capsulitis (6–8 weeks).

In this pilot, 187 patients were referred by prescribing physicians and 145 (78%) initiated digital therapy, completing an average of 3.5 sessions per week and reporting a mean satisfaction score of 9.2/10, with an average reduction in pain intensity of about 40% from baseline and two thirds achieving no functional disability compared with 58% with moderate-to-high disability at baseline. The cohort included a substantial proportion of older adults and people with excess weight: 69% were older than 45 years, 14% were older than 65, and 70% had an above-normal body mass index (21). Patient selection required adequate digital literacy, absence of central sensibilization pain syndromes, cognitive dysfunction, significant visual impairment, or cardiopulmonary disease precluding moderate-intensity exercise. This design corresponds to a before–after, single-arm real-world evaluation, which does not allow robust conclusions about comparative effectiveness, since no control group was defined. These results should therefore be interpreted as suggestive and preliminary evidence, and they motivate further controlled studies to determine whether age, obesity, or other factors meaningfully affect adherence or outcomes. However, the observed results are substantial and warrant further investigation through controlled comparative studies. While direct comparative effectiveness data are lacking, these outcomes support the feasibility of AI-enabled telerehabilitation solutions for appropriately selected patients, particularly in expanding access to those facing geographical or logistical barriers to in-person care.

Evidence from recent systematic reviews shows that while older adults may face specific challenges in adopting digital health technologies, well-designed interventions with intuitive interfaces, perceived clinical usefulness and appropriate training can overcome barriers, enabling health gains in ageing and multimorbid populations (23, 24).

From an economic standpoint, the DE-SNS report provides preliminary cost data based on a comparative, but non-randomised, analysis of different delivery models within the Portuguese NHS. In that pilot, the average cost per patient treated in contracted private PRM clinics is estimated at €307 per completed rehabilitation episode, excluding transport costs, while the average cost per ambulatory patient treated in hospital-based PRM services ranges from €929 (hip arthroplasty) to €1,300 (shoulder procedures) (21). These figures are aggregate episode-level estimates, which limit external validation and comparison with the official national MFR fee schedule^1^. For this reason, the present article uses these values to illustrate the potential order of magnitude of cost differentials and focuses primarily on relative percentage differences. In the telerehabilitation pilot, the average cost per treated episode was €275, already including all direct charges associated with the digital solution, implying a reduction of more than 10% compared with contracted private clinics and substantially larger differences (around 70–80%) relative to hospital-based ambulatory rehabilitation (21). When transport costs and patient time are also considered, the report estimates an additional saving of roughly 10% at the societal level, reflecting avoided travel, reduced productivity losses, and less reliance on hospital-centric pathways.

The cost figures are aggregate episode-level estimates from the DE-SNS pilot. They illustrate order of magnitude differentials rather than precise tariff comparisons and should not be interpreted as formally audited national costs. Critically, cost reduction alone is not the rehabilitation objective. The goal is to maintain clinical outcomes and improve access, whilst using resources efficiently. Economic findings require prospective health economic evaluation and comparative effectiveness studies before informing scale-up policy. These figures illustrate two dimensions. First, from the payer (NHS) perspective, AI-enabled telerehabilitation can lower the direct cost per episode while preserving or improving access and patient-reported outcomes. Second, from a broader societal perspective, additional efficiencies arise through reductions in logistics and transport, avoidance of repetitive and ineffective treatments, and potential productivity gains (9, 21). It is also relevant to consider the fact that the systematic acquisition of objective patient outcome data, such as clinical indicators and patient-reported outcomes, is widely regarded by the WHO and OECD as a quality, efficiency, and equity enabler (25, 26). International evidence shows that integrating such data through digital health infrastructures facilitates precise clinical monitoring, supports adaptive and value-based care, and enables detailed population health analysis (25–28). Addressing this deficit through digital health and AI-enhanced data strategies is recognised as a necessary step towards informed decision-making, accountability and effective public health policies (29, 30).

## The potential for digital disruption and cost optimization

Scaling AI-enabled telerehabilitation nationally could offer a valid alternative for selected patients, providing a more accessible and convenient option while maintaining clinical effectiveness. AI-enabled rehabilitation is not suitable for all clinical situations, particularly when patients require manual techniques, close monitoring, or frequent therapeutic adjustments. In line with WHO guidance and the UEMS-PRM Guidelines and the European PRM White Book (31), rehabilitation must remain medically led, with PRM specialists responsible for diagnosis, functional assessment, prescription, monitoring and management of complications. Experience from the Portuguese pilot reflects this principle: although many patients benefited from digital or hybrid pathways, a proportion, especially in post-surgical programs and subacute pathologies, required conversion to face-to-face care. These findings reinforce that AI-enabled telerehabilitation can serve as a valuable complementary option for appropriately selected patients, while PRM specialists ensure personalized treatment whenever needed.

From a health-system perspective, the scale of current expenditure on contracted rehabilitation suggests that even modest efficiency gains could be meaningful. From the total €180 M spent on Physical Rehabilitation services through conventions with private providers (32) in 2024, approximately €90-126 M were estimated to be due to MSK-related conditions under the 50–70% MSK range described. These figures are scenario-based approximations that depend on assumptions about diagnostic case-mix. It is neither realistic nor desirable to assume that all of this activity could shift to AI-enabled home-based care. Instead, AI-supported telerehabilitation should be seen as a partial substitute for in-person treatment in clearly defined patient and care pathways. To illustrate the potential order of magnitude, consider scenarios in which 50–90% of MSK-related conventioned expenditure could be amenable to some degree of reconfiguration through AI-enabled programmes. Applying this range to the estimated €126 M MSK expenditure in 2024 yields a reconfigurable share of roughly €63 M (50%) and €113 M (90%).

Any estimate of cost must distinguish between different analytic perspectives. From the Portuguese NHS (payer) perspective, the relevant comparison is the direct cost per treated episode: contracted clinic rehabilitation for MSK patients has an average cost of around €307 per episode (excluding transport), whereas the AI-enabled telerehabilitation pilot reported an average cost of €275 per episode, already including all direct charges associated with the digital solution. Under this payer perspective, the focus is on the relative difference in the average cost per treated episode. In the DE-SNS pilot, the reported unit cost of AI-enabled telerehabilitation was approximately 10% lower, already including all direct charges. Applying a similar 8–12% cost differential to the share of conventioned MSK expenditure that could plausibly be reconfigured suggests that, if replicated at scale, AI-enabled programmes could generate meaningful efficiency gains on the portion of activity that transitions to hybrid or home-based digital care, even though the exact budget impact in million euros will depend on the costing methodology and mix of providers. However, the €307 estimate for contracted care and the €275 figure for the telerehabilitation pilot are not derived from an analysis of individual MFR procedures using the national fee schedule. Instead, they are aggregate episode-level estimates specific to the DE-SNS evaluation, and the report does not clarify key costing assumptions. As a result, these values cannot be directly reconciled with the official MFR price list and should not be interpreted as audited national tariffs, but rather as context-specific pilot estimates.

From a broader societal perspective, additional gains arise from reduced logistical and transport needs, facilitate earlier discharge and diminish productivity losses for working-age individuals. Official NHS data on waiting times for PRM consultations show median delays of around 28–63 days for very high-priority cases and 40–120 days for priority cases, rising to 90–248 days for non-priority referrals, with regional variation (33). In contrast, the Portuguese pilot reduced the average time to therapy initiation from more than 60 days to 2.4 days, illustrating how such solutions can expand access (21). However, by lowering access barriers, such models may also generate some degree of induced demand, partially offsetting savings.

The estimates are therefore exploratory, built on a set of explicit assumptions: (i) MSK conditions account for roughly 50–70% of conventioned rehabilitation expenditure; (ii) between 50 and 90% of this MSK activity could be partially reconfigured through AI-enabled solutions for suitable patients; (iii) unit-cost differentials similar to those observed in the Porto pilot are achievable at scale; and (iv) upfront and fixed costs for AI platforms are not yet fully incorporated into the quantitative estimates and may moderate net savings, especially in the early phases of implementation. These limitations reinforce the need for prospective economic evaluations, sensitivity analyses, and rigorous real-world studies before drawing definitive conclusions about the budget impact of national scale-up.

Looking beyond direct costs, the persistent gap in healthy life years at age 65 in Portugal underscores the importance of improving functional outcomes across the life course. National policy frameworks increasingly recognize that digital and AI-driven rehabilitation solutions can contribute to this goal by expanding access, accelerating treatment pathways, and supporting more sustainable and value-based models of care. For non-financial impacts, AI-based solutions can significantly increase access for communities lacking rehabilitation facilities and may contribute to reduced work absenteeism and improve productivity (34). These dimensions further strengthen the case for carefully designed, equity-oriented AI-enabled rehabilitation policies, supported by robust governance and continuous evaluation (35).

## Conclusion

AI-enabled rehabilitation, with home-based prescription and medical supervision, has become a strategic priority for the Portuguese National Health Service, driven by rising demand, access constraints, and the need for sustainable care models. AI-supported telerehabilitation offers a viable solution, with strong evidence demonstrating improvements in access, reduced waiting times, continuity of care, flexibility, system productivity, while aligning with clinical effectiveness, patients satisfaction and the national goals for modernization and health equity.

The successful deployment of such care solutions implies robust governance, specific financing lines, professional training, ethical oversight, data protection, and strong clinical supervision, together with policies that ensure digital inclusion and prevent inequities. With coherent national digital health strategies and evaluation of clinical effectiveness, the integration of certified and strongly tested AI-powered rehabilitation tools can support timely and sustainable rehabilitation care in Portugal, regarding international standards and long-term public health objectives.

## Data Availability

The original contributions presented in the study are included in the article/supplementary material, further inquiries can be directed to the corresponding authors.

## Footnotes

1 According to an ACSS report on ambulatory Physical and Rehabilitation Medicine (PRM), in 2015 the Northern Regional Health Administration recorded an average amount billed per PRM requisition of €142.05 and an average of 2.16 requisitions per user, implying an average expenditure per treated user close to €307 (36).
